# Autophagy and IL-1 Family Cytokines

**DOI:** 10.3389/fimmu.2013.00083

**Published:** 2013-04-05

**Authors:** James Harris

**Affiliations:** ^1^Faculty of Medicine, Nursing and Health Sciences, Monash Medical Centre, Monash UniversityClayton, VIC, Australia

**Keywords:** autophagosome, autophagy, cytokines, inflammasome, inflammation, interleukin, IL-1, IL-18

## Abstract

Autophagy is an important intracellular homeostatic mechanism for the targeting of cytosolic constituents, including organelles, for lysosomal degradation. Autophagy plays roles in numerous physiological processes, including immune cell responses to endogenous and exogenous pathogenic stimuli. Moreover, autophagy has a potentially pivotal role to play in the regulation of inflammatory responses. In particular, autophagy regulates endogenous inflammasome activators, as well as inflammasome components and pro-IL-1β. As a result, autophagy acts a key modulator of IL-1β and IL-18, as well as IL-1α, release. This review focuses specifically on the role autophagy plays in regulating the production, processing, and secretion of IL-1 and IL-18 and the consequences of this important function.

## Introduction

Autophagy exists in three forms. Microautophagy describes the direct engulfment of small volumes of cytosol by lysosomes (Ahlberg et al., 1982). Alternatively, in chaperone-mediated autophagy specific proteins are recognized by a cytosolic chaperone and targeted to the lysosome (Dice, 1990). This review will focus on macroautophagy (hereafter referred to as *autophagy*), a highly conserved homeostatic mechanism for the lysosomal degradation and recycling of cytosolic components, including macromolecules and organelles (Shintani and Klionsky, 2004). Macroautophagy is characterized by the formation of an isolation membrane, or phagophore, which elongates around its target and fuses with itself to form a double-membraned autophagosome. This can then fuse with lysosomes to form an autolysosome, leading to degradation of the luminal contents.

Autophagy acts as an important survival mechanism, sequestering and degrading damaged/toxic cytosolic constituents, such as dysfunctional mitochondria or peroxisomes. In addition, autophagy regulates energy and nutrient homeostasis and plays an essential role in development (Yang and Klionsky, 2010). Autophagy can be induced by numerous different stimuli, including environmental and cellular stresses, such as nutrient deprivation/amino acid starvation, growth factor withdrawal, and endoplasmic reticulum (ER) stress (Lum et al., 2005; Ogata et al., 2006; Yorimitsu et al., 2006).

Autophagy can also regulate a number of important immune responses, including clearance of intracellular bacteria (Deretic, 2010), antigen presentation (Munz, 2010), and the regulation of cytokine production and secretion (Harris, 2011). In addition, autophagy is important for immune cell homeostasis; deficiencies in the autophagy pathway cause defects in ER and leave T cells more prone to cell death (Jia and He, 2011; Jia et al., 2011) and is required for immunoglobulin production by plasma cells (Pengo et al., 2013). Moreover, autophagy in thymic epithelial cells facilitates the presentation of endogenous self-antigens and is thus important for central CD4^+^ T cell tolerance (Aichinger et al., 2013).

Importantly, autophagy is induced by numerous immune stimuli, including exogenous pathogen-associated molecular (PAMPs), such as LPS (Xu et al., 2007), as well as endogenous damage-associated molecular (DAMPs), including HMGB1 (Tang et al., 2010a,b). Moreover, cytokines can regulate autophagy; IFN-γ, TNF-α, IL-1α, and IL-1β all induce autophagy in macrophages (Gutierrez et al., 2004; Harris and Keane, 2010; Shi and Kehrl, 2010), while IL-4, IL-13, and IL-10 have all been shown to inhibit autophagy in macrophages (Harris et al., 2007, 2009; Van Grol et al., 2010; Ní Cheallaigh et al., 2011; Park et al., 2011).

## Autophagy Regulates IL-1α, IL-1β, and IL-18 Secretion

Interleukin 1 family cytokines include IL-1α, IL-1β, IL-18, IL-33, IL-36, IL-37, and IL-38 and orchestrate a wide range of immune and physiological roles. In particular, IL-1α, IL-1β, which signal through the same receptor (IL-1R1), have strong pro-inflammatory effects, largely through the induction of cyclooxygenase type 2 (COX-2), type 2 phospholipase A, and inducible nitric oxide synthase (iNOS) (Dinarello, 2002) and are responsible for the recruitment of myeloid cells, including neutrophils, to sites of inflammation (Rider et al., 2011). IL-18 is similarly pro-inflammatory and both IL-1β and IL-18 are tightly regulated; they are produced as inactive pro-forms that are cleaved by caspase-1 to form the mature, bioactive, cytokines. Caspase-1 is itself activated by an inflammasome, a large multimeric structure that includes an intracellular sensor, such as the NOD-like receptor (NLR) NLRP3 or the DNA sensor, absent in melanoma 2 (AIM2) (Davis et al., 2011). IL-1α, while active in its pro-form, has recently been shown to be more potent as a granzyme B-cleaved truncated peptide (Afonina et al., 2011). Recently, studies have suggested that IL-1β can drive the release of both IL-1α and IL-23 (Harris et al., 2008; Fettelschoss et al., 2011), further highlighting the importance of this cytokine in regulating inflammatory responses.

**Figure 1:**
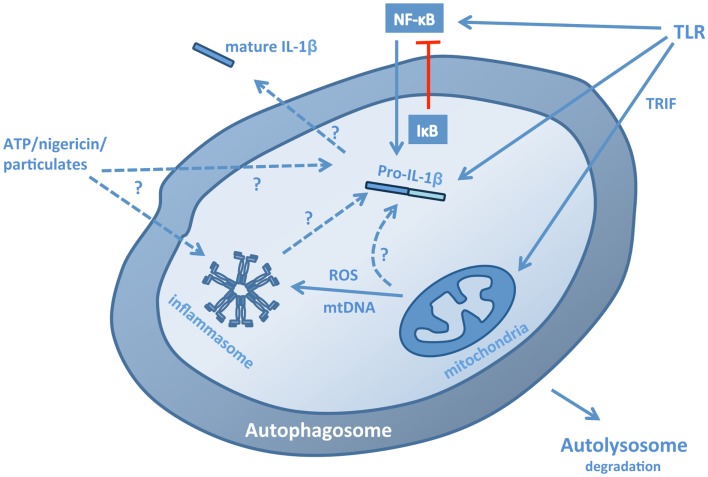
**Pathways for the regulation of IL-1β by autophagy**. Autophagy regulates IL-1β production, processing, and secretion through a number of mechanisms. In the absence of autophagy, stimulation of macrophages or dendritic cells with TLR3 or TLR4 ligands leads to a TRIF-dependent, mitochondrial ROS/DNA-dependent increase in the production, processing, and secretion of IL-1β, suggesting that autophagy normally limits the presence of these stimuli in the cytosol. These stimuli induce inflammasome assembly, but may also increase transcription of pro-IL-1β. Conversely, induction of autophagy in cells stimulated with TLR ligands leads to the sequestration and lysosomal degradation of pro-IL-1β, thus limiting the availability of the cytokine for subsequent processing and secretion. In addition, the inflammasome components ASC, NLRP3, and AIM2 can also be sequestered into autophagosomes. The effects of autophagy on transcription of pro-IL-1β are complex: autophagy down-regulates p62, which may be required for NF-κB activation, but also down-regulates IκB, promoting NF-κB nuclear translocation. Induction of autophagy in the presence of inflammasome-activating stimuli, such as ATP, nigericin and particulates, and crystals, can lead to increased secretion of IL-1β suggesting that autophagosomes may act as part of an exocytic pathway and possibly also a platform for inflammasome assembly, although it is not yet clear whether fully assembled inflammasomes are sequestered.

Studies have demonstrated that autophagy can regulate the transcription, processing, and secretion of IL-1β, as well as the secretion of IL-1α and IL-18 (Figure 1; Table 1). This occurs through at least two distinct mechanisms. Firstly, in macrophages and dendritic cells, inhibition of autophagy, either pharmacologically with 3-methyladenine (3-MA) or through siRNA deletion of autophagy genes, leads to increased release of IL-1β, IL-1α, and IL-18 in response to TLR3 or TLR4 agonists (Saitoh et al., 2008; Harris et al., 2011; Nakahira et al., 2011; Zhou et al., 2011a). Typically, the release of IL-1β is a two stage process. First, transcription of pro-IL-1 is induced by inflammatory stimuli (such as LPS). This is followed by activation of inflammasome assembly by a second stimulus, such as reactive oxygen species (ROS), ATP, particulates (e.g., silica, alum), protein aggregates, or lysosomal disruption. Thus, the inhibition of autophagy results in the accumulation of a second, endogenous, inflammasome-activating stimulus. The second mechanism is more direct; autophagosomes can sequester and degrade inflammasome components, including the adaptor molecule apoptosis-associated speck-like protein containing a CARD (ASC), AIM2, and NLRP3 (Shi et al., 2012), as well as pro-IL-1β(Harris et al., 2011). Studies have not yet addressed whether autophagy might regulate other IL-1 family cytokines, including IL-33, IL-36, and IL-37, but this would be of considerable interest given role in mediating the production and release of IL-1 and IL-18, as well as other cytokines (Saitoh et al., 2008; Crisan et al., 2011; Harris et al., 2011; Nakahira et al., 2011).

**Table 1:** **Interactions between autophagy and IL-1 family cytokines**.

Interaction	Notes	Reference
IL-1α and IL-1β induce autophagy	Induction of autophagy by IL-1 family cytokines suggests a potential negative feedback loop for the control of inflammation by autophagy, as well as a possible anti-microbial response mediated by inflammatory cytokines	Shi and Kehrl (2010)
Inhibition of autophagy increases IL-1α, IL-1β, and IL-18 secretion	Inhibition of (or deficiency in) autophagy leads to increased secretion of IL-1 family cytokines in response to TLR ligands and *Mycobacterium tuberculosis*. This is dependent on TRIF (in mice), reactive oxygen species, and mitochondrial DNA. These results suggest that autophagy normally regulates endogenous factors that would otherwise induce inflammasome assembly, caspase-1 activation, and subsequent processing and secretion of IL-1β and IL-18. In humans, regulation of IL-1β by autophagy occurs at the transcriptional level	Saitoh et al. (2008), Crisan et al. (2011), Harris et al. (2011), Kleinnijenhuis et al. (2011), Nakahira et al. (2011), Zhou et al. (2011a)
Induction of autophagy reduces IL-1β secretion *in vitro* and *in vivo*	Induction of autophagy with drugs (e.g., rapamycin) or starvation reduces secretion of IL-1β by macrophages and dendritic cells in response to LPS with alum or ATP or in response to *Mycobacterium tuberculosis*. Induction of autophagy also decreases intracellular levels of pro-IL-1β. In a mouse model of LPS-induced sepsis, rapamycin decreases serum levels of IL-1β.	Crisan et al. (2011), Harris et al. (2011), Shi et al. (2012)
Autophagosomes sequester and degrade IL-1β and inflammasome components	In macrophages stimulated with TLR ligands, autophagosomes sequester IL-1β. The inflammasome component ASC is ubiquitinated in response to inflammasome activation and delivered to autophagosomes. NLRP3 and AIM2 are also sequestered by autophagosomes	Harris et al. (2011), Shi et al. (2012)
Activation of the NLRP3 and AIM2 inflammasomes induces autophagosome formation	Induction of the AIM2 inflammasome by transfection of macrophages with poly(dA:dT) or the NLRP3 inflammasome with uric acid crystals or nigericin leads to an increase in autophagosome formation	Shi et al. (2012)
Autophagy can act as secretory pathway for the release of IL-1β	Induction of inflammasome activation in parallel with autophagy induction can lead to an increase in IL-1β secretion. This novel secretory pathway is dependent on inflammasome assembly, Atg5, Rab8a, and GRASP55 (GORASP2; a mammalian Golgi reassembly stacking protein paralog)	Dupont et al. (2011)

## A Role for Mitochondria

Two studies have addressed the role of mitochondria in driving the release of IL-1β by autophagy-compromised cells. Zhou et al. (2011a) demonstrated that inhibiting the sequestration of mitochondria by autophagosomes (mitophagy) with 3-MA leads to the accumulation of damaged, ROS-producing mitochondria, which in turn activates the NLRP3 inflammasome, leading to the processing and release of IL-1β. Similarly, Nakahira et al. (2011) established that depletion of the autophagy proteins beclin 1 or LC3B in macrophages leads to the activation of caspase-1 and the release of IL-1β and IL-18 by promoting the accumulation of dysfunctional mitochondria. Moreover, secretion of both cytokines was dependent on mitochondrial DNA (mtDNA), which translocated to the cytosol, a process dependent on ROS and NLRP3. These data would suggest that mitochondrial dysfunction represents an endogenous stimulus for inflammasome activation. Studies have demonstrated that enhanced IL-1β release by autophagy-deficient mouse cells is dependent on TIR-domain-containing adapter-inducing interferon-β (TRIF), an adaptor molecule involved in TLR3 and TLR4 signaling (Saitoh et al., 2008; Harris et al., 2011). However, the role of TRIF in this response is not yet known. In addition, autophagy-deficient NLRP3^−/−^ dendritic cells are still able to secrete IL-1β in response to LPS (albeit at much lower levels than wild type control cells) (Harris et al., 2011), suggesting that other inflammasomes may be activated by mitochondrial instability, at least in mice.

Inhibition of autophagy with 3-MA has also been shown to limit IL-1β transcription in humans through a process independent of caspase-1 activation (Crisan et al., 2011; Kleinnijenhuis et al., 2011). While the mechanism underlying these observations is not yet clear, Lee et al. (2012) have demonstrated that autophagy down-regulates p62, which is important for IL-1β signaling and activation of NF-κB, which leads to increased IL-1β production. However, this is potentially complicated by a number of studies that have demonstrated that autophagy is required for NF-κB activation, as autophagosomes target ubiquitinated IκB for degradation, allowing increased nuclear transcription of NF-κB (Meng and Cai, 2011; Criollo et al., 2012; Jia et al., 2012). Interestingly, in mice, inhibition of autophagy, either with 3-MA or by siRNA targeting of beclin 1, had no effect of IL-1β transcription (Peral de Castro et al., 2012), suggesting important differences between mice and humans in the mechanism through which autophagy regulates IL-1β.

## Autophagy and the Inflammasome

While inhibition of autophagy leads to increased release of IL-1β and IL-18 (as well as IL-1α) in response to TLR3 or TLR4 ligands, induction of autophagy with the mTOR inhibitor rapamycin can inhibit IL-1β release in response to LPS with alum or ATP (Harris et al., 2011). Moreover, activation of IL-1β transcription with TLR agonists, in the absence of an inflammasome-inducing signal, leads to sequestration and degradation of pro-IL-1β by autophagosomes. This process is independent of TRIF (Harris et al., 2011) and would suggest that autophagy acts a self-regulatory mechanism for the control of inappropriate and potentially deleterious inflammatory responses. More recently, Shi et al. (2012) have demonstrated that activation of the NLRP3 and AIM2 inflammasomes induces autophagy in macrophages. Inhibition of autophagy with 3-MA increased inflammasome activation, while induction of autophagy with rapamycin or amino acid starvation limited it. Moreover, inflammasome components, including AIM2, NLRP3, and ASC partially co-localized with GFP-LC3 (an autophagosomal marker) and LAMP-1 (a lysosomal marker) (Shi et al., 2012), suggesting that inflammasomes are degraded within autophagosomes. Interestingly, caspase-1 does not co-localize with GFP-LC3 (Harris et al., 2011), suggesting that sequestration of inflammasome components by autophagosomes is a highly specific process. Again, these data suggest that autophagy represents a regulatory mechanism for the control of inflammatory responses in macrophages.

A recent report has demonstrated that caspase-11 can contribute to NLRP3-dependent IL-1β secretion in a TRIF-dependent manner in response to Gram-negative bacteria. In this response, TRIF activates caspase-11 via type I IFN signaling, which in turn interacts with the NLRP3 inflammasome to regulate caspase-1 activation (Rathinam et al., 2012). It is not yet clear whether autophagy intersects with this TRIF-dependent pathway to regulate caspase-11-dependent IL-1β secretion.

However, the role of autophagy in the regulation of inflammasome activation may not be quite so straightforward. A recent study has demonstrated that induction of autophagy with mTOR inhibitors or by amino acid starvation can lead to increased IL-1β secretion in response to inflammasome-activating treatments, including LPS with nigericin, alum, or silica crystals (Dupont et al., 2011). This effect was partially dependent on Atg5 and at least one of the two mammalian Golgi reassembly stacking protein paralogs, GRASP55 and Rab8a. In these experiments, autophagy was induced at the same time that the inflammasome-activating stimulus was added. Thus, the role of autophagy in regulating IL-1β secretion may depend on timing and context; in the absence of an inflammasome-activating signal, autophagy may act to remove pro-IL-1β and inflammasome components from the cell, while in the presence of such a signal, autophagy may act as a secretory pathway for IL-1β release.

## IL-1α and IL-1β Induce Autophagy

Numerous cytokines are known to regulate autophagy in macrophages, including IFN-γ (Gutierrez et al., 2004), TNF-α (Harris and Keane, 2010), IL-10 (Van Grol et al., 2010; Park et al., 2011), IL-4, and IL-13 (Harris et al., 2007). Amongst those that have been shown to activate autophagosome formation are IL-1α and IL-1β (Shi and Kehrl, 2010; Peral de Castro et al., 2012). Moreover, other cytokines associated with inflammatory responses, including IL-23, have been shown to drive autophagy (Peral de Castro et al., 2012). Thus autophagy may represent an important mechanism in a negative feedback loop to control the secretion of inflammatory cytokines.

## Further Consequences of Autophagic Regulation of IL-1: Effects on IL-23 and IL-17

The regulation of IL-1β release by macrophages and DC subsequently affects IL-23 secretion by the same cells; inhibition of autophagy with 3-MA or by depletion of beclin 1, leads to an increase in IL-23 secretion, driven directly by IL-1β, while induction of autophagy with mTOR inhibitors reduces IL-23 secretion (Peral de Castro et al., 2012). Together, IL-1 (α or β, or IL-18) and IL-23 potently induce the secretion of IL-17 by Th17 cells and innate γδ T cells (Sutton et al., 2009; Mills et al., 2013). Thus, supernatants from LPS-stimulated autophagy-deficient dendritic cells and macrophages, high in IL-1α, IL-1β, IL-18, and IL-23, have been shown to stimulate the secretion of IL-17 by T cells, predominantly γδ T cells (Peral de Castro et al., 2012). This is also relevant *in vivo*, as mice lacking the autophagy protein Atg5 in myeloid cells secrete higher levels of IL-1α, IL-12p70, CXCL1, and IL-17 in response to infection with *Mycobacterium tuberculosis* (Castillo et al., 2012).

## Autophagy and Inflammation *in vivo*

In humans, single nucleotide polymorphisms (SNPs) in the autophagy-related protein 16-like 1 (*atg16l1*) gene have been linked with increased susceptibility to Crohn’s disease (Hampe et al., 2007; Rioux et al., 2007), while Mice lacking Atg16L1 in hematopoietic cells are more susceptible to dextran sulfate sodium (DSS)-induced colitis (Saitoh et al., 2008). Similarly, polymorphisms in the genes encoding other autophagy-related proteins, including Atg2a, Atg4a, Atg4d, Immunity-related GTPase M (IRGM), and ULK-1, have also been associated with Crohn’s disease (Craddock et al., 2010; Henckaerts et al., 2011; Brinar et al., 2012). IRGM is a known modulator of autophagy in human macrophages (Singh et al., 2006), while the mouse ortholog, Irgm1 (formerly LRG-47), modulates IFN-γ-induced autophagy (Gutierrez et al., 2004). Moreover, polymorphisms in IRGM have been linked to the multifactorial autoimmune disease systemic lupus erythematosus (SLE) (Zhou et al., 2011b), as have polymorphisms in Atg5 and Atg7 (Harley et al., 2008; Gateva et al., 2009; Han et al., 2009; Zhou et al., 2011b). In mice with a conditional deletion of Atg7 in the intestinal epithelium, LPS induces higher levels of IL-1β mRNA, compared to wild type controls (Fujishima et al., 2011), while LC3B^−/−^ mice produce higher levels of IL-1β and IL-18 in response to LPS- or cecal ligation and puncture (CLP)-induced sepsis (Nakahira et al., 2011). Moreover, autophagy has a role to play in obesity-related inflammation in mice and humans. Expression of LC3 is higher in the subcutaneous adipose tissues of obese mice and humans, compared to lean controls and correlated with systemic insulin resistance and adipose tissue inflammation (Jansen et al., 2012). In addition, inhibition of autophagy with 3-MA increased the expression of IL-1β, IL-6, and IL-8 mRNA in human adipose tissue explants and IL-1β, IL-6, and keratinocyte-derived chemoattractant (KC) mRNA in mouse explants and this effect was greater in samples from obese individuals/animals (Jansen et al., 2012).

## Conclusion

Autophagy is a highly conserved and ubiquitous process that has many roles to play in cellular homeostasis. Amongst these is the regulation of inflammatory responses to both pathogenic and endogenous stimuli. In particular, autophagy modulates the transcription, processing, and secretion of IL-1β, acting as an important negative feedback mechanism for the control of inflammatory responses, both *in vitro* and *in vivo*. As such, autophagy may represent a potent target for novel anti-inflammatory therapeutics.

## Conflict of Interest Statement

The authors declare that the research was conducted in the absence of any commercial or financial relationships that could be construed as a potential conflict of interest.
